# Impact of Neuronal Membrane Damage on the Local Field Potential in a Large-Scale Simulation of Cerebral Cortex

**DOI:** 10.3389/fneur.2017.00236

**Published:** 2017-06-07

**Authors:** David L. Boothe, Alfred B. Yu, Pawel Kudela, William S. Anderson, Jean M. Vettel, Piotr J. Franaszczuk

**Affiliations:** ^1^U.S. Army Research Laboratory, Aberdeen Proving Ground, Aberdeen, MD, United States; ^2^Altus Engineering, Churchville, MD, United States; ^3^Department of Neurosurgery, The Johns Hopkins University School of Medicine, Baltimore, MD, United States; ^4^The Johns Hopkins Institute for Clinical and Translational Research, Baltimore, MD, United States; ^5^Psychological & Brain Sciences, University of California, Santa Barbara, CA, United States; ^6^Department of Engineering, University of Pennsylvania, Philadelphia, PA, United States; ^7^Department of Neurology, The Johns Hopkins University School of Medicine, Baltimore, MD, United States

**Keywords:** local field potential, Cerebral Cortex, Modeling and simulation, traumatic brain injury, neuronal network

## Abstract

Within multiscale brain dynamics, the structure–function relationship between cellular changes at a lower scale and coordinated oscillations at a higher scale is not well understood. This relationship may be particularly relevant for understanding functional impairments after a mild traumatic brain injury (mTBI) when current neuroimaging methods do not reveal morphological changes to the brain common in moderate to severe TBI such as diffuse axonal injury or gray matter lesions. Here, we created a physiology-based model of cerebral cortex using a publicly released modeling framework (GEneral NEural SImulation System) to explore the possibility that performance deficits characteristic of blast-induced mTBI may reflect dysfunctional, local network activity influenced by microscale neuronal damage at the cellular level. We operationalized microscale damage to neurons as the formation of pores on the neuronal membrane based on research using blast paradigms, and in our model, pores were simulated by a change in membrane conductance. We then tracked changes in simulated electrical activity. Our model contained 585 simulated neurons, comprised of 14 types of cortical and thalamic neurons each with its own compartmental morphology and electrophysiological properties. Comparing the functional activity of neurons before and after simulated damage, we found that simulated pores in the membrane reduced both action potential generation and local field potential (LFP) power in the 1–40 Hz range of the power spectrum. Furthermore, the location of damage modulated the strength of these effects: pore formation on simulated axons reduced LFP power more strongly than did pore formation on the soma and the dendrites. These results indicate that even small amounts of cellular damage can negatively impact functional activity of larger scale oscillations, and our findings suggest that multiscale modeling provides a promising avenue to elucidate these relationships.

## Introduction

Human cognitive performance relies critically on cerebral cortical electrical activity within the 1–100 Hz frequency range (1–5), and *in vivo* neural activity can be measured as potential differences in the electric field which manifest itself as current in a recording electrode (1). In humans, electrical activity is commonly recorded as electroencephalography (EEG), where electrodes are placed on the outside of the head/skull, or electrocorticogram, where electrodes are placed directly on the cortical surface of the brain. Both of these electrophysiological monitoring methods record the integrated activity of millions of neurons simultaneously, and any disruption to neurons that degrades large-scale brain activity within these frequency ranges has the potential to impair cognitive performance (6, 7). Directly observing how damage at the neuronal level links to impairments in cognitive performance is not possible given the current state of *in vivo* human neuroimaging (8–11). Consequently, large-scale simulation modeling provides a means to work around the limitations of *in vivo* imaging since the impact of neuron-level damage on network activity can be tracked as changes in model activity (12).

Following blast events, the connection between physical damage mechanisms and mild traumatic brain injury (mTBI) cognitive impairments has been inconsistent (13, 14). Initial success in understanding the biological substrate of severe traumatic brain injury has focused on morphological changes such as diffuse axonal injury and gray matter lesions that can be observed using magnetic resonance imaging (13, 14). However, less debilitating forms of traumatic brain injury, such as acute concussion, are not normally associated with large-scale structural damage (13), but research has shown that disruption of microscale neuronal processes can impact whole-brain electrical activity (15, 16). Our simulations explore the possibility that negative effects of mTBI could be due to microscale neuronal damage occurring across many neurons simultaneously, and it is their cumulative impact that disrupts coordinated cortical oscillations.

A number of studies have reported observing blast-induced damage to the neuronal membrane, either directly (17–22) or through damage to proteins known to maintain membrane structural integrity (14, 23, 24). Similarly, simulations of molecular damage following blast exposure have predicted the appearance of ion permeable pores on the surface of the neuronal membrane (25, 26). These results imply that a blast could induce significant impairment in the regulation of ionic flow across the membrane, which is necessary for critical electrophysiological functions such as action potential (AP) generation. Complementary research revealed that exposing *in vitro* nervous tissue directly to blast can induce acute changes in electrical activity in the absence of cell death, including three primary effects on *in vitro* hippocampal electrical activity: decreased neuronal synchrony, decreased neuronal firing, and decreased neuronal excitability (27). One plausible mechanism for the negative impact on electrical activity within these experiments is that blast causes a disruption of the neuronal membrane sufficient to alter electrical activity but not sufficiently strong to cause cell death.

Here, we explored how cellular damage to neurons alters collective electrical activity that may have an impact on cortical function. This approach requires the use of detailed and physiologically realistic model neurons connected together to simulate a patch of cortical tissue that generates an output which can be compared to experimentally recorded activity. Our model was an adaptation of a previously published thalamocortical network model (28, 29) modified to be used in the GEneral NEural SImulation System (GENESIS) neuronal simulation environment (30). Neurons were modeled using multiple compartments in order to generate realistic electrical activity in the 1–100 Hz frequency range known to be relevant for capturing task-relevant brain activity (1, 2).

Given their role in neuronal communication, we hypothesized that damage to the axons may have more substantial effects on connections between simulated cortical patches than damage to the soma and dendrites. To emulate cellular damage following a blast event, we simulated the formation of pores on the membrane by changing the membrane conductance to mimic the expected increase in ionic flow across the membrane. We parameterized the amount of reduction in membrane resistance to provide a scalar quantity to estimate varying levels of damage to the cell, and then neuronal functional activity was assessed using APs at the cellular level and local field potentials (LFPs) at the larger scale for coordinated activity among distributed cortical patches of neurons. The LFP signal is thought to be the basis of the electrical signals (EEG) and magnetic field fluctuations (MEG) recorded at the scalp that has been linked to task performance, so alterations in LFP activity serves as a proxy for potential decrements in cognitive performance following neuronal injury.

## Materials and Methods

### Individual Neuron Model

Simulated neurons in our model are biophysically detailed with each neuron being represented by multiple compartments and voltage dependent channels. Using biophysically detailed simulated neurons allows the model to generate realistic electrical activity as an emergent property of the underlying physics (1). Neurons were simulated with 50 to 137 compartments representing somata, axons, and apical, basal, and distal dendrites (28, 29). Each compartment is represented by a differential equation for membrane potential where each channel is treated as a separate conductance (31). Differential equations were solved using the exponential Euler method at a 50 µs time step. Passive current properties within and between compartments were modeled via cable equations (32). Electrical activity within each neuronal compartment was simulated using up to 15 distinct voltage-gated channels representing calcium, potassium, and sodium ion dynamics. The model contains 14 neuron types organized into six cortical layers. Excitatory cells included are: layer 2/3 regular spiking and fast rhythmic bursting pyramidal cells, layer 4 stellate cells, layer 5 tufted intrinsic bursting and tufted regular spiking pyramidal cells, and layer 6 non-tufted pyramidal cells. Inhibitory cells included are layer 2/3 superficial basket interneurons, superficial axo-axonic interneurons, and low threshold spiking interneurons; and in layer 5, deep fast-spiking basket interneurons, deep axo-axonic interneurons, and deep low threshold spiking dendrite connecting interneurons. Thalamic cells included were both the inhibitory nucleus reticularis thalami and excitatotory thalamocortical relay cells. Our model is designed to produce resting cortical electrical activity in the absence of sensory inputs, so cortical layer 4 pyramidal cells have not been included. Excitatory neurons are glutamatergic and associated with AMPA and NMDA postsynaptically, while inhibitory neurons are associated with ionotropic GABA_A_ synapses.

### Simulating Blast-Induced Membrane Damage

Nanoscale modeling of blast impact on the membrane of neurons found that microscale pores appeared on the surface of the neuronal membrane that were sufficiently large to allow the flow of ions through the membrane (26). Other simulations have shown that increasing levels of biaxial stretch induced on a simulated neuronal membrane can increase both the number and the size of ion permeable pores (25). One expects for the appearance of even a small number of pores in the neuronal membrane to have a relatively large impact on ionic flow and generate a commensurate reduction in the electrical resistance of the neuronal membrane (*R*_m_). Since the exact relationship between the number and size of membrane pores and changes in electrical resistance of the membrane has not been experimentally determined, we performed a parameter sweep to determine how changes in membrane resistance impact overall model electrical activity. We report these results as the level of damage to the neuronal membrane, and this terminology references a scalar quantity that represents the amount of reduction in membrane resistance where 10% cell damage corresponds to a simulation using a *R*_m_ at 90% of the baseline, whereas 50% cell damage corresponds to *R*_m_ at 50% of its baseline value. Changes in membrane resistance were made at equal percentage levels simultaneously across all simulated neurons.

Additionally, while there is a wealth of experimental evidence describing large-scale blast-induced damage to axons (in the form of white matter tracts (33–36)), less is known about the impact of blast on either the soma or dendrites of neurons (17). In order to determine whether there were location-dependent impacts on electrical activity, we simulated damage to the whole cell, the axons alone, and the somatic and dendritic compartments.

### Cortical Patch Model

Adapted for the GENESIS simulation environment, our model was based on a previously published thalamocortical network model (28, 29). All parameter values used in the simulations are identical to the published description (29), except for three modifications. First, as described in the Section “Simulating Blast-Induced Membrane Damage,” we modified the resistance of the neuronal membrane to simulate varying levels of damage. Second, we removed simulated gap junctions to capture neuronal activity of healthy individuals as our baseline. Gap junctions are thought to have a role in generation of high frequency epileptic seizure activity in adult cerebral cortex (29) and impact neuronal connectivity and synaptogenesis in the neonatal cortex (37), but their role in generation of normal baseline brain activity is poorly characterized at the present time (38–40). Thus, we eliminated gap junction so we could examine how simulated damage impacted otherwise healthy functional activity. Finally, the neuronal morphologies were converted to three dimensions so that the model had a more realistic spatial representation of electrical activity, resulting in random neuron placement in the vertical and horizontal planes but within the limits of the proper layer of cortex.

**Figure 1:**
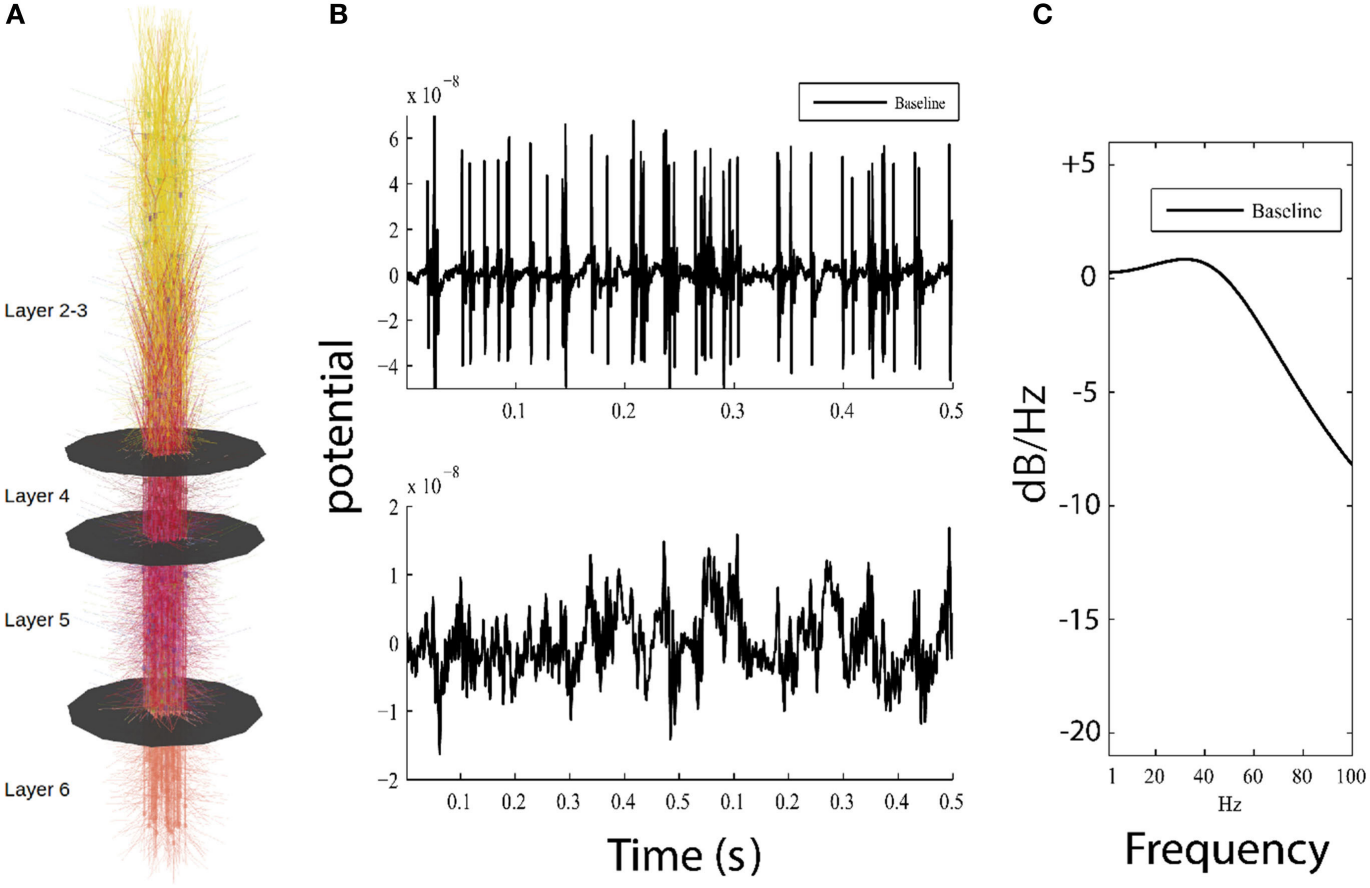
Overview of model and simulation output. **(A)** Visual representation of the model, showing 585 cortical neurons and layer boundaries depicted as black disks. **(B)** Top panel shows 0.5 s of high-pass filtered model output for the baseline model used for detection of action potentials, while bottom panel plots the low-pass filtered model output simulating local field potential which resembles low frequency patterns seen in electroencephalography (EEG). **(C)** Parametric power spectrum estimate shows power in logarithmic scale (normalized to 0 dB/Hz at −190 dB/Hz) where higher frequencies have less power than lower frequencies; this result approximates 1/*f*, which is similar to power observed in neuroimaging methods (electrocorticogram and EEG).

Our cortical network represents a 150 µm × 150 µm × 2,871 µm patch of cortex along with corresponding thalamic relay cells and downstream thalamic targets (Figure 1A). Connection strength between axons and synaptic targets were weighted randomly and subject to distance-dependent exponential decay. Synaptic dynamics are represented as activation currents scaled by the maximal synaptic conductance and the connection weight. Connection probabilities ranged from *p* = 0.01 to.25, except in the 36 inhibitory axo-axonic interneurons which were fully connected to the axon initial segments of all pyramidal cells and layer 4 spiny stellate cells. In our model, a total of 335,067 connections were simulated between 585 neurons, and neurons were organized into microcolumns containing 23 or 24 neurons each. Each microcolumn contains each of the 14 neuron types described in the Section “Individual Neuron Model,” with multiple identical pyramidal cells being simulated in layers 2/3, 5, and 6 of each microcolumn. Axonal conduction delays were proportional to the distance between the soma of the presynaptic neuron to the postsynaptic compartment. APs were transmitted between simulated neurons as all-or-none messages from the soma of the firing cell directly to their synaptic targets on destination cells with a distance-weighted conduction delay (1 s/m). To simulate resting neuronal activity, in each simulation, we drove 10% of the neurons with independent Poisson distributed excitatory postsynaptic potentials (λ = 0.005) representing uncorrelated background activity from other brain regions. All simulations used an identical random seed making their connectivity and the number, timing and location of the Poisson distributed excitatory postsynaptic potentials identical for all model runs.

### Simulated Electrical Activity

The membrane potential of each compartment in our model was simulated using a sampling rate of 20 kHz (dt = 50 µs), allowing us to explore both intrinsically generated APs from the individual neurons and the LFP across a group of neurons. The first second of simulated data was excluded to allow the model to settle from initial conditions. Data were then processed using the MATLAB^®^ signal processing toolbox.

Action potentials were obtained by resampling the output to 5,000 Hz and then high-pass filtering the data using a ninth order Butterworth filter with a cutoff frequency of 50 Hz (Figure 1B, top). The number of APs per simulation was determined using a membrane potential threshold of 2.5 × 10^−8^ V. The mean AP firing rate was then calculated as the total number of APs across all simulated neurons divided by the length of the simulation time.

For the LFP measurement, raw model activity was recorded at the center of the simulated patch of cortex as the summed distance-weighted membrane potential of all simulated compartments. The recording electrode was placed at a sufficient distance perpendicular to the model surface (*z*-coordinates) to keep distance-dependent effects to a minimum. LFPs were then obtained by resampling the membrane potentials from the model to 1,000 Hz and then low-pass filtering using an eighth order Butterworth filter with a roll off of 300 Hz (Figure 1B, bottom).

### Power Spectral Density and Statistical Methodology

Local field potential power spectral density (Figure 1C) and its 99% confidence intervals were estimated using Burg’s method (MATLAB^®^ function pburg). The model generates a stationary LFP time series from our relatively short (20 s) simulation runs, and the parametric spectral estimator allows us to obtain a smooth and consistent power estimate (41). The Akaike information criterion calculated for all simulated LFPs showed that a fourth order model provides the best compromise between under and overfitting for all investigated conditions. Assigning model order using Burg’s method is analogous to fitting data using a polynomial, where a higher model order reflects a larger number of separable behaviors in the data. For each level of simulated damage to the model, the ratio of power in damaged cells to baseline power was calculated, and this difference is plotted in logarithmic scale in 1–100 Hz frequency range to illustrate the damping effect of decreased ionic flow across the membrane.

Confidence intervals were obtained by the resampling method embedded in the MATLAB^®^ pburg function. These confidence intervals were confirmed using asymptotic methods. Additionally, we were interested in whether or not overall power changed across specific frequency bins, so we calculated power over the frequency ranges of interest as the sum of power spectral density at 0.1 Hz intervals from 1 to 40 Hz (390 points) and from 40 to 100 Hz (600 points). The variance of the overall power estimate was calculated as the sum of the variances of the individual power spectral density values. The significance of the difference between power estimates for baseline and simulated cell damage for each of the ranges and damage levels was then obtained from Student’s *t* cumulative distribution using the MATLAB^®^ function tcdf. Finally, Bonferroni correction was applied to account for multiple comparisons.

## Results

Our primary aim was to examine whether simulated cellular damage that mimicked membrane pore formation after blast could induce electrophysiological changes that negatively impact brain electrical activity in the absence of either cell death or large-scale structural lesions. We first examined the impact on functional activity following damage to the whole cell, and then we investigated whether this impaired functional activity was differentially modulated by damage to axons compared to damage to somas and dendrites.

### Simulated Damage to the Membrane of the Whole Cell Reduces AP Generation

**Figure 2:**
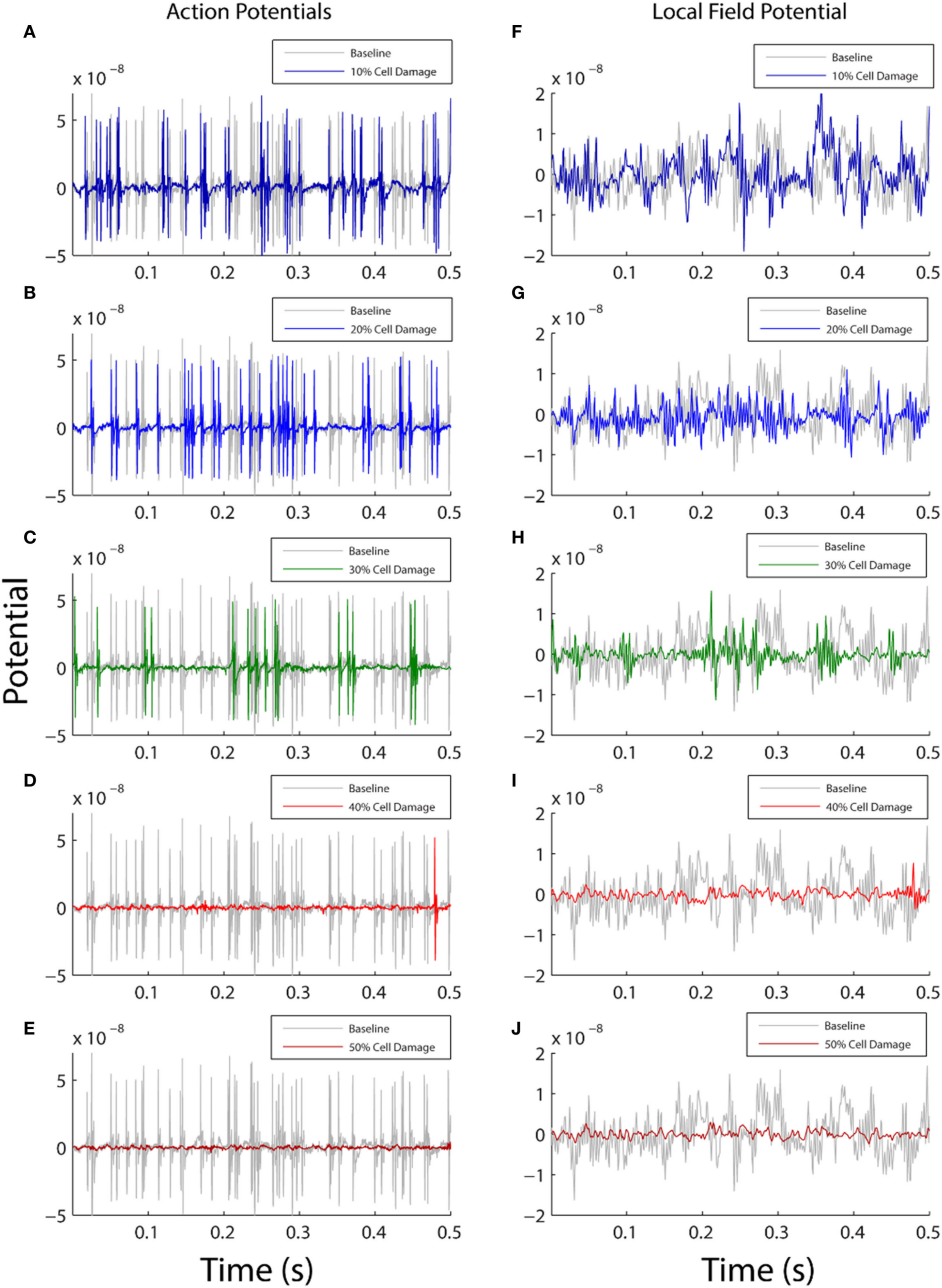
Comparison between baseline model output (gray) and damaged model output (color). The left column shows action potential generation **(A–E)** and the right plots local field potential from the model **(F–J)**. Each row shows how the model activity changes as a function of varying the membrane resistance with 10% damage in top row and 50% damage in bottom row. On the *y* axis, membrane potential is shown in arbitrary units, and the *x* axis shows simulation time for the first 0.5 s of model data (total simulation time 20 s). In both columns, the amount of simulated functional activity decreases as the amount of simulated cellular damage increases.

In the first analysis, we parametrically modified the membrane resistance on the entire surface of the neuronal membrane (whole cell) to examine the effect of ionic flow disruption on functional neuronal activity. We expected that increasing membrane damage would reduce activity across all frequencies equally, given the broadband nature of the change (across all compartments equally) and that the reversal potential of the neuronal membrane is in the inhibitory range since it is closer to the reversal potential of potassium than it is to the reversal potential of sodium [*V*_M_ = −70 mV, *V*_K_ = −95 mV, and *V*_Na_ = +50 mV (29)]. Our results demonstrated this expected effect: increasing levels of damage to the neuronal membrane were associated with both a decrease in neuronal firing rates (Figures 2A–E and Table 1, row 1), and an overall reduction in the amplitude of the simulated LFP (Figures 2F–J).

**Table 1:** Rate of action potential (AP) generation at varying levels of membrane damage (columns) and different location of damage (rows).

Baseline = 79 APs/s	10% Damage	20% Damage	30% Damage	40% Damage	50% Damage
Whole cell	69 APs/s	55 APs/s	34 APs/s	7 APs/s	0 APs/s
Axons	75 APs/s	65 APs/s	55 APs/s	42 APs/s	24 APs/s
Soma and dendrites	77 APs/s	71 APs/s	66 APs/s	56 APs/s	36 APs/s

*Damage to whole cell causes the greatest reduction in neuronal firing rates, while damage to axons causes a greater reduction in neuronal firing than damage to the soma/dendrites*.

### Simulated Damage Reduces LFP Power in the 1–40 Hz Range More Strongly Than in the 40–100 Hz Range

**Figure 3:**
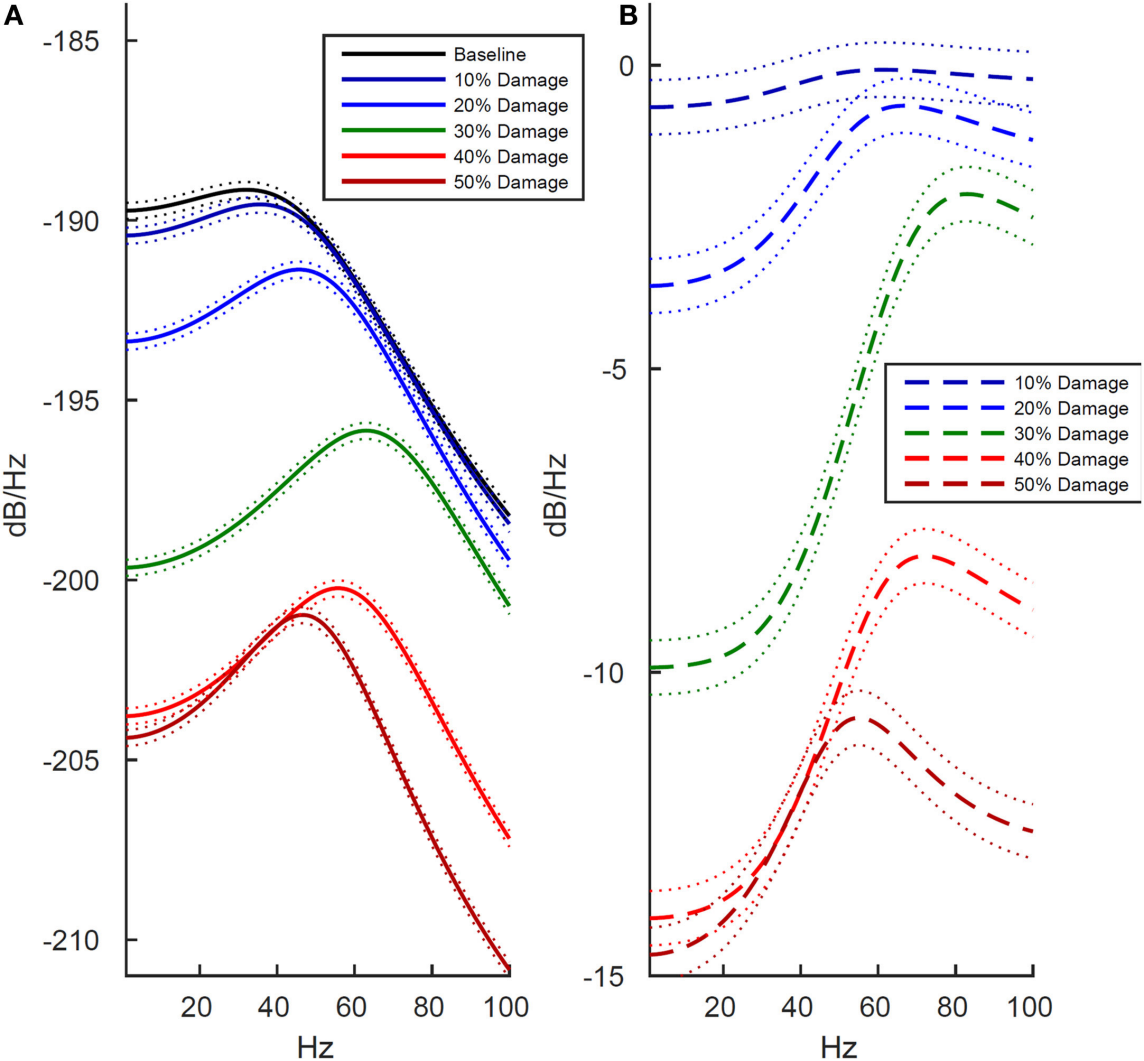
Effects of cell membrane damage on spectral power of simulated local field potential. Across both plots, colored lines represent different levels of damage to the cell membrane (black is baseline and damage increases from blue to red in increments of 10%), and dotted lines indicate 99% confidence intervals. **(A)** Effect of damage to the whole cell. **(B)** Difference in log-scaled spectral power between baseline (undamaged) and simulated damage to the whole cell. As expected, power is reduced at higher levels of simulated damage, but all frequencies are not effected equally. In the lower frequencies, ranging from 1 to 40 Hz, there is drop in power of approximately 14 dB/Hz between the 10 and 50% levels of damage. Likewise, in the 40–100 Hz range power drops 12 dB/Hz.

We next examined how these changes in the LFP activity manifested in spectral power estimates. The reduction in LFP amplitude was confirmed as a reduction in power across the entire 1–100 Hz frequency range, shown in both the power estimate from the damaged model (Figure 3A) and the power estimate difference between the damaged model and baseline model (Figure 3B). Unexpectedly, the reduction in amplitude within the LFP was more pronounced within the lower frequency ranges. As shown in Figure 3, at the 30% damage level, the largest drop in power was 10 dB/Hz in the 1–40 Hz low frequency range (*p* < 0.01), while the largest drop in power was 6 dB/Hz in the 40–100 Hz frequency range (*p* < 0.01). At the 50% damage level, the reduction in power between low and high frequency ranges were similar with low frequency (1–40 Hz) power exhibiting a largest drop of 15 dB/Hz (*p* < 0.01) confidence interval, and high frequency (40–100 Hz) exhibiting a largest drop of 11 dB/Hz (*p* < 0.01). To put these values in context, an attenuation of 3 dB at a given frequency reflects a drop to half power from maximum. Damage at the 50% level was also associated with an absence of neuronal firing (Table 1, row 1, column 5), and this resulted in the minimal LFP power with activity generated from only subthreshold neuronal membrane fluctuations Simulated damage to axons negatively impacts functional activity more than damage to soma and dendrites.

### Simulated Damage to Axons Negatively Impacts Functional Activity More Strongly Than Damage to Soma and Dendrites

To better understand the origin of these LFP effects after whole cell damage, we investigated whether the decreased functional activity was differentially modulated by damage to axons than damage to somas and dendrites. We investigated this by running separate simulations. In the first, we selectively damaged the membrane in simulated axons while leaving the soma and dendrites intact, and then in a second set of simulations, we damaged the somal and dendritic membranes while leaving the axons intact.

**Figure 4:**
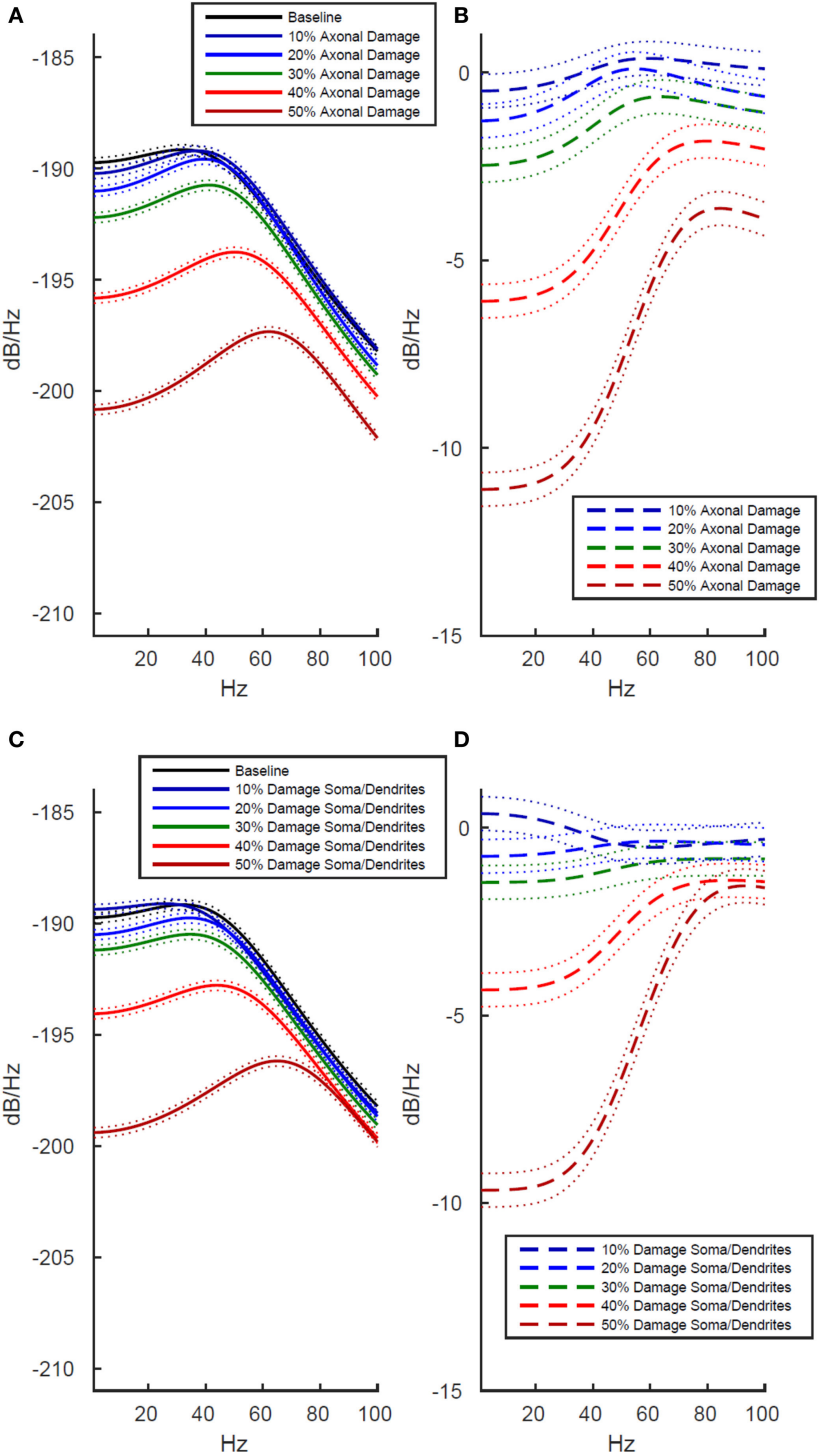
Effects of localized membrane damage on spectral power of simulated local field potential. Dotted lines indicate 99% confidence interval. Damage is modeled as a reduction in membrane resistance localized to either simulated axons or simulated somas and dendrites. **(A)** Effect of selective damage to only the axon compartments of the cell. **(B)** Reduction in power from baseline for axonal damage. **(C)** Effect of selective damage to the soma and dendrite compartments of the cell. **(D)** Reduction in power from baseline for damage to somal and dendritic compartments of the model. Greater functional effects are observed after axonal damage, particularly in the low frequency range where power is reduced to a greater extent than in the high frequency range.

As shown in Figure 4, we found that damage to axons reduced model LFP power more strongly than damage to the soma/dendrites. There are two interesting exceptions to this trend. First, in the low frequency range at minimal damage levels (10%), damage to the soma and dendrites actually led to slightly increased power, while an identical level of damage to the axons decreased power over the same frequency range (Figures 4B,D, dark blue line). Second, in the high frequency range at 10–20% damage levels, damage to soma/dendrites results in a stronger reduction in LFP power than damage to axons.

The location specificity of membrane damage found in LFP was confirmed in an analysis of AP production. As shown in Table 1 (rows 3 and 4), damage to the axons always reduces mean AP firing rates more than damage to the soma/dendrites. For instance damage to the axons reduces firing rates by 2 APs/s more than damage to the soma and dendrites at the 10% level of damage and by 12 APs/s at the 50% damage level (Table 1).

### General Patterns of Simulated Damage on Neuronal Activity

Finally, we directly compared the power spectrum plots across all three tested conditions (whole cell, axons, soma and dendrites) to identify global patterns of simulated damage on neuronal activity. Results revealed that damage at the 10% level results in only small changes from baseline, but when simulated damage increases to 20–30%, damage to the entire cell is greater than the sum of damage to the axons and the soma and dendrites taken together (Figures 3 and 4, *p* < 0.01 at 99% confidence interval). Finally, at maximum levels of damage, the LFP power in the low frequency range hits the minimum value at 40% damage, while LFP power in the higher frequency range does not bottom out until 50%.

## Discussion

In this research study, we created a multiscale brain model to investigate the structure–function relationship between cellular changes at a lower scale and coordinated oscillations at a higher scale, and we capitalized on results within the literature on cellular changes found after blast events. Unlike severe traumatic brain injuries that led to structural lesions detectable by neuroimaging methods, mild traumatic brain injuries often led to performance decrements that have been hard to link directly to structural or functional damage that can be imaged *in vivo* in humans. Here, we used a physiology-based model of cerebral cortex built in the GENESIS simulation framework to explore the possibility that performance deficits characteristic of blast-induced mTBI may reflect dysfunctional, local network activity influenced by microscale neuronal damage at the cellular level.

Our physiology-based model of cerebral cortex consisted of 14 neuron types organized in six cortical layers, and each cortical neuron had its own compartmental morphology and electrophysiological properties. We operationalized microscale damage to neurons as the formation of pores on the neuronal membrane based on the blast literature, and we simulated pore formation by varying the membrane resistance to create varying levels of simulated damage, ranging from 10 to 50%, in increments of 10. We compared the functional activity of neurons before and after simulated damage and found that simulated pores in the membrane reduced both AP generation and LFP power in the 1–40 Hz range of the power spectrum, a range also implicated in research on task performance. Damage to the axons also resulted in stronger effects than damage to the soma and dendrites, both in terms of reduced LFP power and decreased AP generation. Critically, all of these negative impacts would be possible in the absence of cell death or structural lesions that are detectable with current neuroimaging methods. Furthermore, the statistical significance between damage conditions in this simulated data suggest that the effects are robust enough to be visible even in experimental measurements with additional noise and/or artifact sources of signal based on the narrow confidence intervals for power estimates and high significance of the calculated differences in power. Thus, our modeling results suggest that even small amounts of cellular damage can negatively impact functional activity of larger scale oscillations, and our findings indicate that multiscale modeling provides a promising avenue to elucidate these relationships.

### Simulating the Impact of Blast As Changes in Membrane Resistance to Reflect Pore Formation

The closest point of experimental and theoretical contact between pore formation and changes to the electrical resistance of the neuronal membrane comes from research on electroporation. Electroporation is a technique used to create pores in cellular membranes to increase molecular permeability. Simulations have shown that both electroporation and blast are likely to induce pores in the neuronal membrane of similar surface area [~1 nm^2^ (25, 42, 43)], and that this pore size is sufficiently large to allow for ions to travel across the membrane [an area of greater than 0.0785 nm^2^ (44)]. Blast-induced pores would not need to cover a large portion of the neuronal membrane in order to significantly reduce membrane resistance since pores of less than 1 nm^2^ comprising approximately.003% of the total membrane area are predicted by simulation to reduce the overall resistance of the neuronal membrane by approximately 4 orders of magnitude (44). Additionally, in electroporation drops in membrane resistance of up to 300% have been observed in the absence of significant cell death (45). Although the mapping between electroporation and blast-induced pore formation is not well studied, these experimental findings suggest that the range of simulated damage in our modeling effort, including the max value of 50%, is unlikely to cause cell death. Pores formed due to both blast and electroporation have also been shown to occur on similar time scales with both persisting for hours after exposure (21, 45). However, given that the impact of mTBI occurs over time periods of days, weeks, and years, and membrane permeable pores occur on the time scale of hours, it is unknown whether or not blast-induced pores could account for longer term additional, persistent structural or functional damage to the cell.

### Cellular Damage May Underlie Detrimental Changes in Functional Activity

In our simulations, damage to the neuronal membrane caused an overall reduction in neuronal activity, leading to a decrease in LFP power and a reduction in the mean rate of AP generation. While it has been shown that exposure to blast can cause microscale damage to the neuronal membrane (14, 20, 23, 24), it is less clear what the impact of damage to the neuronal membrane would have on coordinated electrical activity between brain regions. Recent experiments in *in vitro* hippocampal slices recorded changes in underlying neuronal network electrical activity which were exposed to blast like pressure waves in the absence of significant cell death (27). This study found several changes in the electrical activity of the brain tissue, including the robustness or amplitude of the experimentally measured electrical response, the amount of stimulation necessary to induce half of the maximal network electrical response, and a coarse measure of neuronal synchrony. Although we did not use external stimulation in our model, our results are comparable since we also found a reduction in neuronal firing rate as well as a reduction in overall LFP amplitude and power after decreasing the membrane conductance.

### Effects of Simulated Damage Are Location Dependent on the Cellular Membrane

In our simulations, the location of neuronal damage had an impact on the severity of the observed reduction in LFP power. Damage to the whole cell caused the greatest disruption of electrical activity, while damage to the axons alone reduced LFP power more than the same amount of damage to the soma and dendrites. Reduction in LFP power when damage occurs equally across all parts of the neuron could not be reduced to a simple linear summation of the effects of damage to either axons or somas and dendrites alone. All three of these components of the neurons within our simulation represent unmyelinated short range connections within a small patch of gray matter. The reduction in simulated LFP power due to changes in the membrane resistance impacting strictly local connections emphasizes that large-scale structural changes may not be necessary to produce noticeable impairment in cortical electrical activity. Given our results, we expect that damage to neurons which impairs local function within the gray matter of the cerebral cortex could make a significant contribution to coordinated network activity, which is likely compromised following an mTBI.

The stronger impact of damage to axons on model network behavior as compared with damage to the soma and dendrites could have important functional consequences for the impact of structural changes on whole-brain electrical activity. Diffuse axonal injury is a classic signature of severe TBI, which is characterized by widespread structural lesions in the white matter fiber tracts that connect brain regions (13, 46). In severe TBI, shearing and deformation forces can immediately injure axons (47), leading to measurable decrements in performance on cognitive tasks (33–36). Our simulations suggest that damage to axonal membrane not visible as structural lesion in white matter may also impact the function of axonal communication between cortical regions. Higher levels of damage may cause severe reduction in AP generation (Table 1), and as the fundamental signaling mechanism of neurons, this functional decrement would impair communication between different cortical regions at any distance.

### Potential Behavioral Consequences of Decreased Functional Activity in the 1–100 Hz Range

Overall, we found that simulated damage to the neuronal membrane significantly reduced model LFP power in the 1–100 Hz frequency range, and this range is associated with a wide variety of cognitive processes (3), including attention and memory (5, 48), decision making and motivation (48), and multisensory integration (4). Similarly, patients with mTBI exhibit symptoms that are highly variable and include anxiety, impaired sleep, concentration difficulties, impaired memory, and headache (49–51). Since clinical mTBI symptoms are so diverse, researchers have begun to focus on discovering general cognitive processes whose disruption could explain mTBI impairment across multiple cognitive domains. One such study found that patients with mTBI had attentional deficits that were not attached to specific sensory modalities (51), while another showed that patients with long term concussive mTBI had both reduced cognitive efficiency and increased fatigability, as well as general deficits in working memory (52). Since brain activity within the 1–100 Hz range is associated with a broad spectrum of cognitive processes, we expect that disruption of brain activity in this frequency range may impact a wide range of brain activity, a finding reflected in the various clinical symptoms across mTBI patients.

Furthermore, our simulations indicate that for weaker levels of membrane damage (10–30%), neuronal activity in the 1–40 Hz range will be more strongly disrupted than higher frequency neuronal activity within the 40–100 Hz range (Figures 3 and 4). This nicely complements previous mTBI studies that have shown that mTBI behavioral impairment was most strongly correlated with changes in EEG activity in the 1–30 Hz frequency range (53). Activity within the 1–40 Hz range measured using quantitative EEG techniques has also been impaired in patients with mild TBI (54). The relatively larger impact of changes in membrane resistance on network electrical activity in the low frequency range allows for the possibility of using EEG as a biomarker for possible mTBI when there is no visible structural damage using other neuroimaging tools since EEG recordings are typically limited to frequencies below 40 Hz due to the attenuation of higher frequencies by the intervening tissue between the cortex and the scalp.

### Modeling Other Damage Mechanisms Using Electrophysiology-Based Models

Many diverse microscale damage mechanisms have been hypothesized to induce mTBI under blast conditions. Although we only directly examined pore formation and decreased membrane resistance, there are many other potential damage mechanisms that would impact model behavior in the ranges we present here, including a negative impact on brain activity in the 1–100 Hz frequency range. Future research could extend our results and investigate additional damage mechanisms with little to no additional variables or parameters in the model, such as damage to synapses, morphological changes, changes in connectivity, ion channel deformations, and changes in calcium regulation. Of particular interest, models could examine ionic conduction through voltage-gated sodium and potassium channels. This conduction critically depends on a highly structured set of charged proteins to be in a specific conformation for their proper function, and disruptions that reduce ionic flow through these channels are implicated in genetic disorders (55) and are likely candidates for damage following blast-induced channel deformations. Damage to voltage-gated channels like this could either lead to epileptic seizure if it involves potassium channels or lead to a reduction in overall levels of activity if it occurs in sodium channels. Similarly, damage to synapses can be simulated as either changes in maximal synaptic conduction, or as a change in the shape of the simulated postsynaptic potential.

In general, damage mechanisms that evolve on a longer time scale are more difficult to simulate without the addition of variables/parameters to standard electrophysiology-based models such as metabolic dysregulation, glial neuron interactions, learning and memory, g-protein-coupled processes, and protein synthesis. It is possible to simulate the end point of many of these damage mechanisms, like changes in ionic balance due to damage to glia, but it is exceedingly difficult to simulate this type of damage over the time course of days, weeks, or even years.

### Conclusion and Future Directions

Our simulations show a relationship between simulated cellular damage and the power spectrum of the LFP. Future work to expand our results could be accomplished by increasing the strength of both inherent model oscillatory activity and observing the influence of external input/stimulation on model behavior. Here, model activity was driven by a random Poisson process, which most closely corresponds to activity in a small patch of cortex in a resting state, or a cognitive state where the brain is not processing any specific external input. This results in simulated activity that does not have strong oscillatory behavior. With higher levels of membrane damage and absence of significant production of APs, the LFP power described here is dominated by subthreshold and independent membrane potential fluctuations. This limits the direct mapping of our results to resting EEG, since the human brain maintains the ability to generate strongly oscillatory activity following mild traumatic brain injuries. Thus, the expansion of our model to include simulated sensory inputs to cortex is a natural next step for our research which will allow for investigating the impact of membrane damage on oscillatory activity related to sensory input processing. More generally, our model provides a framework to understand the relationships between changes in membrane parameters and large-scale activity among cortical brain regions critical for everyday cognition and task performance.

## Author Contributions

All authors contributed equally to the study conception and design. The modeling and simulation was executed by DB, AY, PK, and WA. DB, AY, JV, and PF all contributed to the data analysis and interpretation. The manuscript was written and approved for publication by all authors.

## Conflict of Interest Statement

The authors declare that the research was conducted in the absence of any commercial or financial relationships that could be construed as a potential conflict of interest.
